# Extinctions, genetic erosion and conservation options for the black rhinoceros (*Diceros bicornis*)

**DOI:** 10.1038/srep41417

**Published:** 2017-02-08

**Authors:** Yoshan Moodley, Isa-Rita M. Russo, Desiré L. Dalton, Antoinette Kotzé, Shadrack Muya, Patricia Haubensak, Boglárka Bálint, Gopi K. Munimanda, Caroline Deimel, Andrea Setzer, Kara Dicks, Barbara Herzig-Straschil, Daniela C. Kalthoff, Hans R. Siegismund, Jan Robovský, Paul O’Donoghue, Michael W. Bruford

**Affiliations:** 1 Department of Zoology, University of Venda, Private Bag X5050, Thohoyandou 0950, Republic of South Africa; 2 Konrad Lorenz Institute of Ethology, Department of Integrative Biology and Evolution, University of Veterinary Medicine, Vienna, Austria, Savoyenstr. 1A, 1160 Austria; 3 Cardiff School of Biosciences, Sir Martin Evans Building, Cardiff University, Museum Avenue, Cardiff, CF10 3AX, United Kingdom; 4 National Zoological Gardens of South Africa, 232 Boom Street, Pretoria, 0001, South Africa; 5 Department of Genetics, University of the Free State, 205 Nelson Mandela Drive, West Park, Bloemfontein, 9300 South Africa; 6 Department of Zoology, Jomo Kenyatta University of Agriculture and Technology, Kenyatta Avenue, Nairobi, 00200, Kenya; 7 Department of Biological Sciences, Thomas Building, University of Chester, Chester, CH1 4BJ, United Kingdom; 8 Vienna Museum of Natural History, Burgring 7, 1010, Vienna, Austria; 9 Swedish Museum of Natural History, Frescativägen 40, Stockholm, 10405, Sweden; 10 Department of Biology, University of Copenhagen, Ole Maaløes Vej 5, Copenhagen N, DK-2200, Denmark; 11 Department of Zoology, Faculty of Science, University of South Bohemia, Branišovská 1760, České Budějovice, 37005, Czech Republic

## Abstract

The black rhinoceros is again on the verge of extinction due to unsustainable poaching in its native range. Despite a wide historic distribution, the black rhinoceros was traditionally thought of as depauperate in genetic variation, and with very little known about its evolutionary history. This knowledge gap has hampered conservation efforts because hunting has dramatically reduced the species’ once continuous distribution, leaving five surviving gene pools of unknown genetic affinity. Here we examined the range-wide genetic structure of historic and modern populations using the largest and most geographically representative sample of black rhinoceroses ever assembled. Using both mitochondrial and nuclear datasets, we described a staggering loss of 69% of the species’ mitochondrial genetic variation, including the most ancestral lineages that are now absent from modern populations. Genetically unique populations in countries such as Nigeria, Cameroon, Chad, Eritrea, Ethiopia, Somalia, Mozambique, Malawi and Angola no longer exist. We found that the historic range of the West African subspecies (*D. b. longipes*), declared extinct in 2011, extends into southern Kenya, where a handful of individuals survive in the Masai Mara. We also identify conservation units that will help maintain evolutionary potential. Our results suggest a complete re-evaluation of current conservation management paradigms for the black rhinoceros.

The well documented poaching and subsequent demographic collapse of black rhinoceros (*Diceros bicornis*) populations, including the western subspecies (*D. b. longipes*) declared extinct in 2011, has raised fears that this species will disappear from the wild within the next two decades^1^ (Fig. 1a). During the 20^th^ century, populations are thought to have declined by more than twenty-fold until the mid-1990s, when intensive protection led to a population recovery to just over 5,000 individuals by 2014. Despite a historic range that included much of sub-Saharan Africa (Fig. 1b), the black rhinoceros now survives in only five countries: South Africa, Namibia, Kenya, Zimbabwe and Tanzania (ranked by total population size). Renewed poaching has threatened this recovery, as rhinoceros horn has attained an unprecedented and steadily rising value of $65,000 per kilogram^1^. At the 16^th^ meeting of the Conference of Parties to the Convention on International Trade in Endangered Species (CITES 2013)^2^, it was reported that poaching of black and white rhinoceroses in South Africa had increased from 13 per annum in 2007 to 455 by mid-October 2012, and Zimbabwe, where populations are much smaller, lost an average of 39 rhinoceroses per annum between 2000 and 2011 (CITES 2013)^2^. Recently, it has been reported that rhinoceros poaching has reached a critical point, and if the killing continues, rhinoceros deaths would exceed births in 2016–2018^3^. Annual poaching counts have exceeded 1,000 individuals each year since 2013^4^.

Recently, molecular genetic approaches have been deployed in black rhinoceros conservation for forensic identification and enforcement purposes^5^. However, in contrast, large-scale genetic management of black rhinoceros populations, including the assignment of individuals, their remains and products, to source populations, has been hampered by a lack of a range-wide understanding of the species’ genetic variation. Such information is now crucial to better understand ongoing trade and to monitor remaining populations^6^,^7^.

The lack of a range-wide genetic assessment of *D. bicornis* is problematic because the evolutionary status of black rhinoceros populations has been debated for decades^8^,^9^,^10^,^11^,^12^,^13^,^14^. The four-subspecies classification of du Toit (1987)^11^ dividing the species into the western (*D. b. longipes*), eastern (*D. b. michaeli*), south-central (*D. b. minor*) and south-western (*D. b. bicornis*) subspecies was adopted by the IUCN’s African Rhino Specialist Group (AfRSG) and is the prevailing basis for conservation management of this species (Fig. 1b). However, the geographic and taxonomic patterns used to underpin this scheme coincide with gaps in specimen data, which need to be filled to better define population limits^15^.

During the 20^th^ century, most black rhinoceros populations were hunted to low numbers and many went extinct (Fig. 1a). One reason for the absence of a geographic overview of historic population ranges is that local extinctions, especially those occurring earlier in the 20^th^ century (mainly in West, Central and North-East Africa), may have occurred even before populations were identified by the relevant conservation authorities. In addition, the populations of each of the five countries in which black rhinoceroses still persist are mostly the result of large scale consolidation of fragmented local populations by means of past translocations and it is unknown how these admixed stocks relate to their founding local populations.

Previous black rhinoceros genetic studies have largely been restricted, either in geographic scale, molecular coverage or sampling^16^,^17^,^18^,^19^,^20^ and therefore have not had the power to comprehensively examine the genetic consequences of population extinctions and declines in order to aid management or define conservation units. Genetic extinction, defined by the irrecoverable loss of genetic diversity, may not occur after local population extinction provided that some of the populations survived. Surviving populations undergo an even more serious threat as rapid demographic declines are often accompanied by decreases in effective population size, diminished population genetic diversity and a simultaneous amplification of genetic structure. This manifestation of ‘genetic erosion’^21^, is a process the Convention on Biological Diversity now recognises should be minimised^22^ and can threaten populations where, for example, dispersal options are limited^23^. Therefore, studying the genetics of extant populations is important for conservation monitoring. Understanding the effect of population extinctions and declines can be greatly aided by an assessment of the distribution of historic genetic diversity prior to population collapse^24^,^25^. Analysis of historic samples can clarify the fraction and distribution of pre-decline genetic diversity that remains within extant populations. Furthermore, a geographically comprehensive data set that includes historic material allows for a more accurate assessment of species’ evolutionary and demographic histories.

Molecular data is now routinely used to help define conservation units within threatened species that require management plans. The evolutionarily significant unit (ESU) and management unit (MU) concepts sought to conserve the evolutionary potential of a species by defining populations possessing unique evolutionary and adaptive variation on the basis of phylogenetic distinctiveness and/or differences in allele frequencies, respectively^26^,^27^,^28^,^29^,^30^. Despite its continued use in practical conservation planning, ESUs and MUs have been criticised for, among others, lacking a spatial dimension, which could define the appropriate geographic scale at which population units should be managed^31^. Defining ESUs and MUs in space also allows the incorporation of a degree of natural or directed gene flow in management strategies in cases where conservation units have currently or historically overlapping or adjacent spatial ranges.

We assessed the pre-decline genetic variation of the black rhinoceros across its known historic range using a comprehensive set of museum and modern samples. We used these data to first infer the evolutionary history of the species and then to document how hunting has compressed, partitioned and eroded genetic diversity within and among the species’ final strongholds. Finally, we spatially evaluated our molecular data to determine the geographic components of black rhinoceros molecular diversity, from which we define candidate genetic units for conservation.

## Results

### Mitochondrial and nuclear diversity

Mitochondrial DNA variation was found to be unevenly distributed across the species range. East Africa (Uganda, Tanzania, Kenya) harboured the highest levels of museum sample variation, followed by countries in southern Central Africa (Zambia, Malawi, Angola & Zimbabwe; Table 1). In general, diversity was lower towards the limits of the species’ range in West, North-East and South-West Africa. Of the 20 countries where black rhinoceros samples could be obtained, aboriginal populations of black rhinoceroses persist today in only five (Table 1).

**Figure 2:**
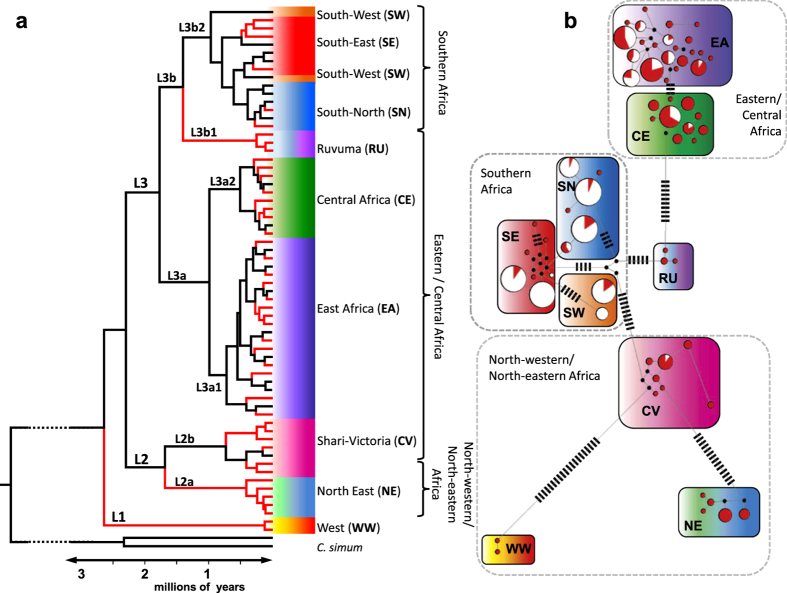
Phylogenetic and network analyses. (**a**) Bayesian phylogeny of 64 mitochondrial DNA (mtDNA) control region haplotypes, obtained from a sample of 403 individual sequences. The maximum clade credibility tree was constructed from the combined posterior tree sample of five independent runs. Only branches with a posterior probability of 1 are shown. Lineages are labelled along each branch. Haplotypes coloured in black were isolated from at least one extant individual, whereas haplotypes from red were isolated only from museum samples, assumed extinct and (**b**) median-joining network of 64 mitochondrial DNA (mtDNA) control region haplotypes showing haplogroup relationships. Each white and/or red filled circle denotes a haplotype, the size of each circle is proportional to the frequency at which that haplotype was observed in the data set, and the proportion of red/white fill is the ratio of museum/extant samples belonging to each haplotype. Small black circles denote median vectors. Small black lines denote the number of mutation steps separating distant haplotypes. All other haplotypes are separated by one or two mutations. Both phylogeny and network show support for seven reciprocally monophyletic haplogroups (WW, NE, CV, EA, CE, RU, SN), but spatial structuring increased resolution to nine haplogroups, adding SE and SW. Spatially informed haplogroups are colour coded and superimposed onto both phylogeny and network. WW, west of the Shari-Logone River system; CV, east of the Shari-Logone to East Africa; NE, North-East Africa; EA, East Africa to the Zambezi River; CE, Central Africa to the Zambezi River; RU, Ruvuma region between Kilombero and Shire Rivers; SN, Southern Africa (Northern); SE, Southern Africa (Eastern); SW, Southern Africa (Western).

Of the 64 haplotypes observed in the full dataset, only 20 (31%) could be detected in samples from extant populations (black haplotypes; Fig. 2a), suggesting a major loss of range-wide mitochondrial diversity during the 20^th^ century (red lineages; Fig. 2a, Table 1). This loss was also apparent at the country level. Historic data were available for four of the five countries harbouring extant populations, and all showed a marked decrease in mitochondrial variation. East Africa (Tanzania and Kenya) was most severely affected where the number of haplotypes reduced from 34 to 11. In South Africa six historical haplotypes were reduced to one (Table 1). Only nine haplotypes were detected among extant populations across southern Africa (haplotypes indicated by white circles; Fig. 2b).

Levels of nuclear diversity were also higher in historic (museum) samples. Since pre-existing data sets were incorporated into our study, data from all loci were not available for all samples (Table 2). Although we were able to amplify nuclear loci in fewer (56) museum samples, the expected heterozygosity (*H*_E_) for these samples was the highest recorded for this species (0.75; Table 2). For country and regional comparisons, we only considered populations with five or more samples. The highest levels of historic microsatellite diversity were observed in East Africa (Tanzania and Kenya), followed by southern Central and South-West Africa. Although not significant (*p* = 0.16), decreases in *H*_E_ and the mean number of alleles per locus (A) were detected when historic and modern samples were compared, but this trend was not as marked as for the mitochondrial DNA (mtDNA) dataset.

### Genetic erosion

**Figure 3:**
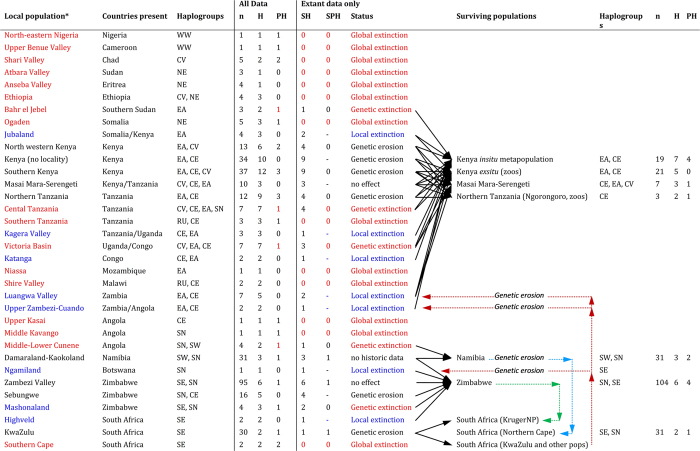
Extinctions and genetic erosion at historic localities across the black rhinoceros’ range and how these map onto surviving populations. n = number of samples, H = number of haplotypes, PH = number of private haplotypes, SH = number of surviving haplotypes, SPH = number of surviving private haplotypes. Localities in red are globally or genetically extinct and those in blue are locally extinct. Only localities in black are still extant. Black arrows map shared haplotypes between locally-defined historic populations and extant populations. Red arrows, genetic erosion by extralimital introduction of the KwaZulu-Natal population; blue arrows, genetic erosion by extralimital introduction of Koakoland-Damaraland population; green arrows, reintroduction of the Zambezi Valley-Sebungwe population to South Africa.

Only nine of the described local populations had not been extirpated in their original habitat, and of these, six were classed as potentially genetically eroded while only two (the Masai Mara, and the Zambezi Valley) were found not to have lost haplotypes (Fig. 3). However, if we consider that the introduction of haplotypes to regions in which they did not historically occur also constitutes genetic erosion, then the surviving KwaZulu-Natal haplotype has been introduced extralimitally to countries where the original black rhinoceros population was hunted to extinction such as Malawi, Zambia and Botswana (dashed red arrows; Fig. 3). Similarly, the introduction of Damaraland-Kaokoland black rhinoceroses from Namibia to the Northern Cape province of South Africa constitutes an extralimital introduction (blue arrows; Fig. 3). On the other hand, some translocations may have been beneficial to the long term population survival. The introduction of both Zambezi Valley-Sebungwe and KwaZulu-Natal individuals to the Kruger National Park in the north-eastern part of South Africa (green arrows; Fig. 3) is genetically compatible, since the Zambezi population historically shared alleles with the KwaZulu-Natal population.

**Figure 4:**
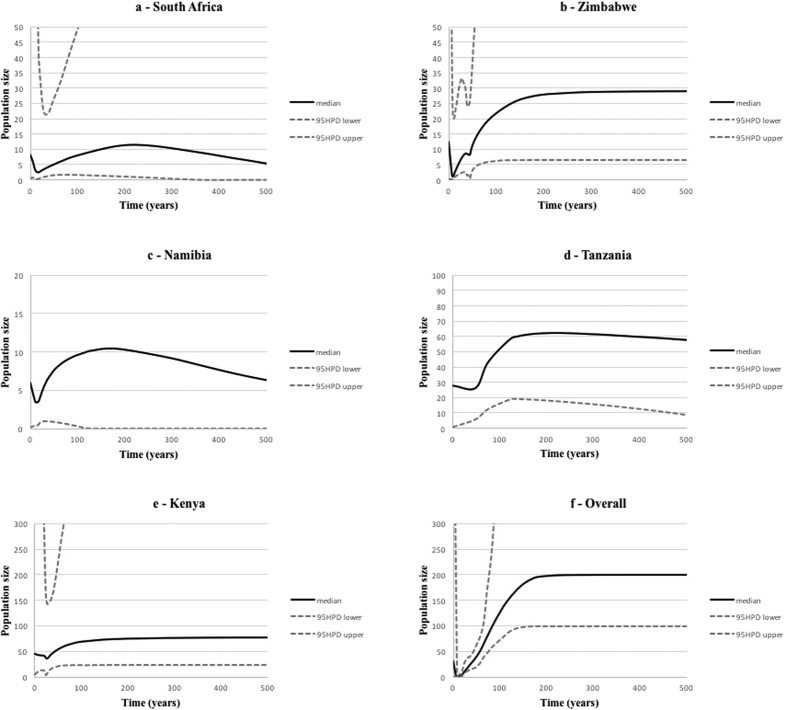
Bayesian skyline method for estimating demographic history from mitochondrial DNA (mtDNA) sequences. (**a**–**f**) Bayesian skyline plots showing changes in effective size over recent time for each of the five extant populations and the species overall. Solid black lines indicate the posterior density of the effective population size (*N*e) estimates, and dashed grey lines indicate the 95% highest posterior density (HPD) intervals. Time in years on the x-axis represents time in years from the present.

We also investigated whether the entire surviving black rhinoceros population and the five remaining country populations have undergone demographic changes as a result of the local and genetic extinctions documented above. BEAST analysis of the mitochondrial control region data set showed clearly that the entire black rhinoceros population has undergone a collapse in effective population size, beginning approximately 200 years ago, and reaching its lowest point some 15 years ago before slightly recovering (Fig. 4a–f). Despite no evidence for the loss of historic haplotypes in Zimbabwe and the Masai Mara Game Reserve, all extant stocks showed trends similar to the global data set, reaching their lowest effective population levels in the latter half of the 20^th^ century, then recovering to between 40–70% of their starting numbers. The highest number of pre-decline effective breeders was Kenya (77), followed by Tanzania (62), Zimbabwe (28), South Africa (11) and Namibia (10).

### Local vs genetic extinctions

The historic data collected for this study was used to compile a geographic overview of local populations prior to 20^th^ century declines. We identified 34 local populations and listed them geographically from West to North-East Africa and then to Central and southern Africa (Fig. 3). Southern Kenya contained the highest number of haplotypes historically, whereas central Tanzania harboured four of the nine mtDNA haplogroups observed in the total data set (Table 1). Eleven local populations were found to be globally extinct in West, North-East, Central and South Africa, where none of their historic haplotypes were observed in extant populations. These global extinctions accounted for half of the loss (22/44) of the mtDNA haplotypes since historic times. Loss of further unique mtDNA variation occurred through five genetic extinctions, where private haplotypes were lost as a result of the extirpation of a local population. In seven cases, the extirpation of a local population did not result in a loss of historic haplotypes as these can still be observed among extant populations in East, Central and southern Africa.

### Mitochondrial and nuclear genetic structure

Phylogenetic reconstruction showed that mitochondrial control region sequence variation was highly structured, comprising three divergent lineages (Fig. 2a), the most distinct of which (L1) comprised two haplotypes sampled only from animals west of the Shari-Logone River system (Haplogroup WW from Nigeria and Cameroon). The other two lineages were broadly divisible into a North-eastern/North-western African lineage (L2), and a subdivided L3 lineage distributed in Central, eastern and southern sub-Saharan Africa. Across the entire species range, seven monophyletic haplogroups (WW, NE, CV, EA, CE, RU and SN), could be identified (see Supplementary Information for a full description of haplogroups and their distribution in subSaharan Africa).

**Figure 5:**
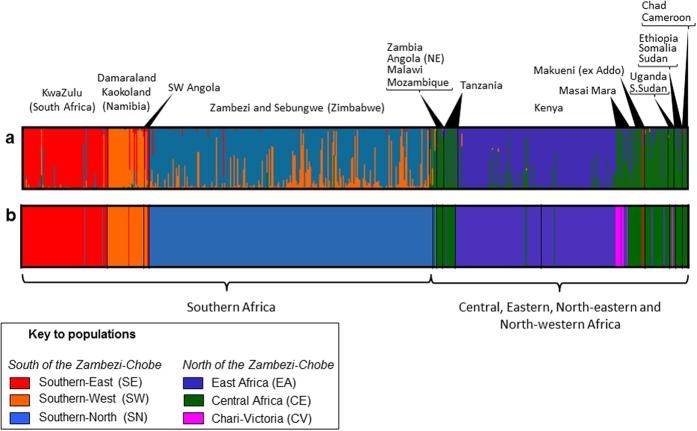
Range-wide nuclear genetic structure of the black rhinoceros. (**a**) Bayesian population assignments carried out from microsatellite data in Structure using the admixture model revealed five population groups and (**b**) spatially informed population assignment carried out in BAPS revealed a sixth population in the Victoria Nyanza Basin. Populations are labelled by extant stock or by country of origin, colour coding is as in Fig. 2.

Nuclear DNA variation was strongly structured and we found that the optimal number of clusters was observed at *K* = 5 (Fig. 5a). While *K* = 5 was not the simulation that resulted in the highest log-likelihood values, it partitioned the data set into the maximum number of biologically meaningful units without the introduction of ghost populations. Clustering assuming *K* = 5 was consistent, with all five replicates inferring the same clustering. In contrast, lower and higher values of *K* introduced minor clusters in some replicates. For a full description see the Supplementary Information.

### Spatial structure across sub-Saharan Africa

**Figure 6:**
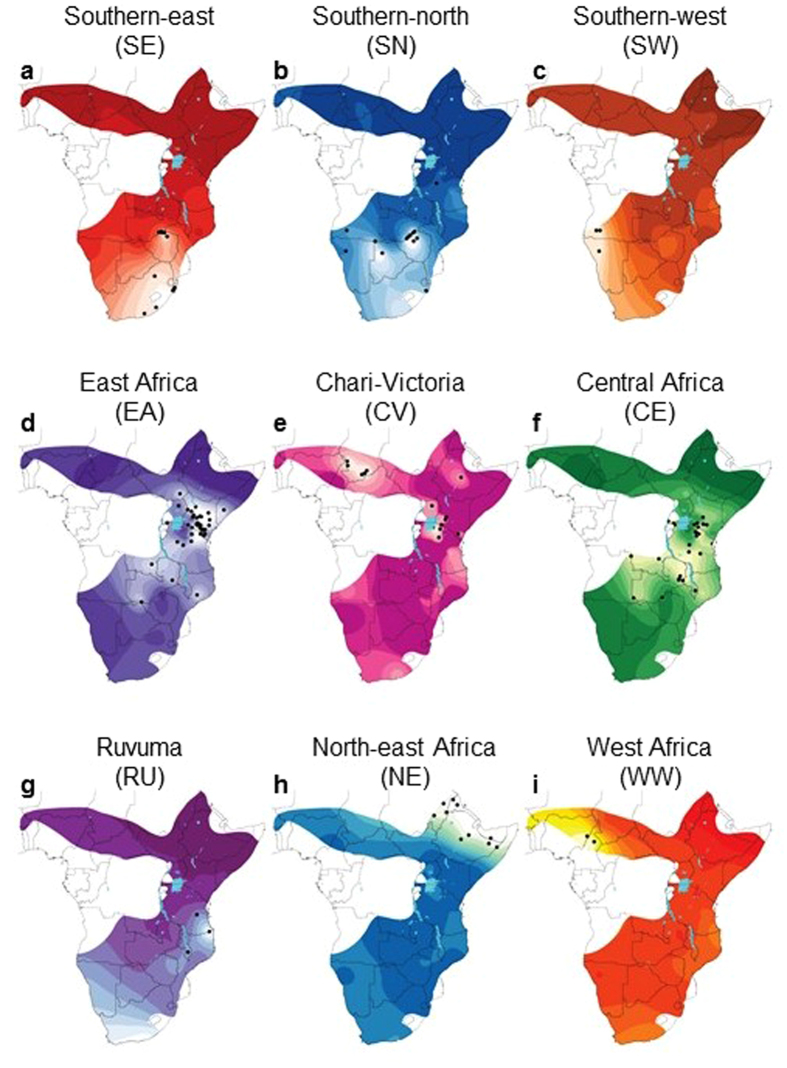
Historic ranges of nine black rhinoceros mitochondrial DNA (mtDNA) haplogroups. (**a**–**i**) Bayesian spatial structure of each haplogroup was interpolated onto separate range-wide distribution maps, thereby defining the historic distributions of each haplogroup. Colour coding of spatially informed haplogroups follows Fig. 2. Small black dots represent the geographic locations where members of each haplogroup were sampled. Lighter colour gradients within the limits of the historical species range, usually lightest around sampling locations, indicate the regions in which each haplogroup is expected to occur at highest posterior probability. Maps were created using the following R packages: geoR^67^, raster^68^, rgdal^69^ and maptools^70^.

**Figure 7:**
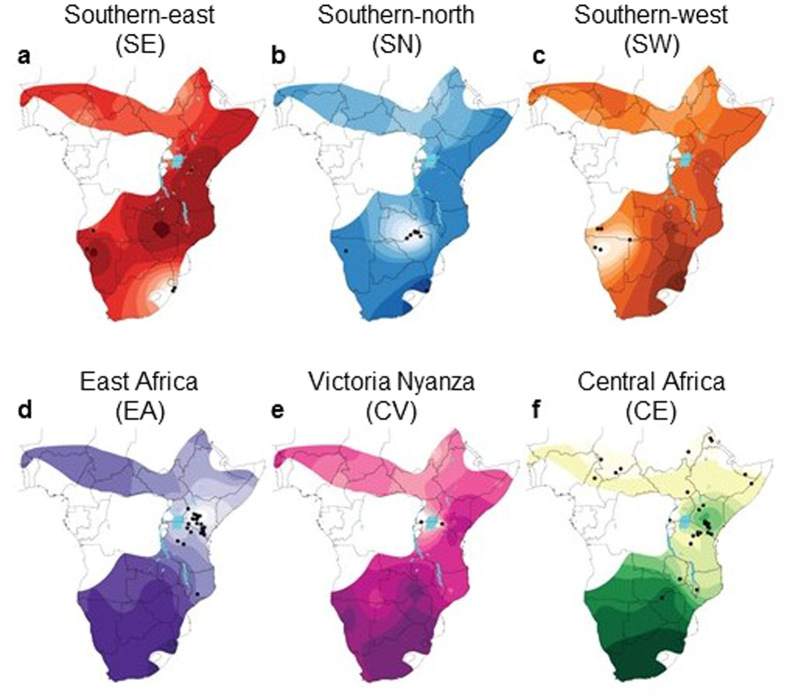
Historic distribution of six nuclear DNA (nDNA) black rhinoceros populations. (**a**–**f**) Structure of each populations was interpolated onto separate range-wide distribution maps, defining the historic distributions of each population. Colour coding follows Fig. 2. Small black dots are sampling locations. Lighter colour gradients within the limits of the historical species range, usually lightest around sampling locations, indicate the regions in which each population is expected to occur at highest posterior probability. Maps were created using the following R packages: geoR^67^, raster^68^, rgdal^69^ and maptools^70^.

The posterior probabilities of population membership were interpolated onto maps to infer the historic distribution of haplogroups. For the mtDNA data set, results were strongly in accordance with the phylogenetic tree and haplotype network (Figs 2a and b, Fig. 6), but with the addition of haplogroups SE and SW, brought the number of mtDNA haplogroups to nine. The haplogroup SW comprises only two lineages, both of which were found exclusvely among either Kaokoland-Damaraland (Namibia) or Middle-Lower Cunene (South West Angola) individuals. We found the most probable number of spatial clusters in our microsatellite data set to be six (Fig. 7). An additional nuclear DNA (nDNA) population from the Victoria Nyanza basin (potentially equivalent to the CV mtDNA haplogroup) was identified on the basis of genetic and geographic exclusiveness (Figs 2a and 5b). Spatial analyses also increased the number of equivalent populations identified by both marker types: SN, SE and SW to the south of the Zambezi-Chobe system and EA, CE and CV to the north of it. Despite their monophyly, and the increased resolution of spatial analysis, the mtDNA haplogroups WW, NE and RU remained unresolved by the microsatellite data.

Overlaps in the spatial boundaries of population/haplogroup distributions were observed in East and southern Africa (Figs 6 and 7). In East and Central Africa, the distributions of the CE and EA haplogroups almost completely overlapped, and although this was also true for the nDNA populations (Fig. 7), the microsatellite data appeared to localise the EA nDNA population to Kenya and northern Tanzania. In southern Africa the distribution of mtDNA haplogroups SN and SE overlapped considerably, with SW remaining geographically distinct. All three nDNA populations SN, SE and SW appeared geographically isolated.

### Conservation units

We used the inferences above to define units for management of the black rhinoceros. Eight haplogroups can potentially be considered ESUs under Moritz’s (1994) criterion^29^ of reciprocal monophyly (Table 3). Of these, however, only four were geographically distinct (WW, NE, RU, SW) and thus qualify as spatially coherent ESUs. Only one population (SE) that differentiated through differences in microsatellite allele frequencies (Fig. 5a and b) was not monophyletic, and therefore qualified as a MU. Two monophyletic haplogroups with overlapping distributions were observed in East Africa (EA and CE). However, since they were sister clades (Fig. 2a), we collapsed them into a higher order ESU and make no inferences on within-ESU structure (Table 3). A similar situation occurred for SN and SE in southern Africa, except that SE was only differentiated by nuclear DNA. The nuclear DNA results showed differences in microsatellite allele frequencies for the populations EA, CE, SN and SE (Fig. 5a).

## Discussion

This is the first study defining the range-wide geographic levels and structure of putatively neutral genetic variation in the black rhinoceros, a highly threatened species for which such an assessment has long been overdue^15^,^32^,^33^. The hitherto unknown information revealed by this study is potentially of critical value for the conservation management and long-term survival of this species since these data revealed populations that have been historically connected and this information can also be used in translocation/reintroduction strategies from wild and captive breeding programs.

This study emphasises the value of historical data in quantifying losses in genetic diversity and inferring effective population size changes across the range of a widely distributed species. The inclusion of historical material where possible can provide opportunities to investigate demographic histories (i.e. effective population size changes) that span large numbers of generations^34^. If this study had been conducted on only extant populations, our inferences would certainly have been misled by the widespread extinction of mtDNA lineages and strong post-decline genetic drift, as found in a recent genetic analysis of Indian tigers^25^. This is clear by examining a phylogeny that could be reconstructed from just extant samples (black lineages; Fig. 2a) which lack 69% of the species’ mtDNA variation. Both markers showed a wide range in the levels of historic genetic diversity for countries in sub-Saharan Africa (Tables 1 and 2) and coalescent simulations using mtDNA for surviving country populations also showed different levels of historical effective population size (Fig. 4a–f). This could suggest histories of differing degrees of population contraction, gene flow and isolation in different parts of the continent.

Despite this, the five countries with surviving populations of black rhinoceroses have undergone marked reductions in effective population size (Fig. 4a–e). Low levels of genetic variation among these surviving populations were a common feature across all loci, suggesting they resulted from demographic (e.g., genetic drift) rather than selective processes. South Africa showed the earliest onset of genetic erosion, inferred as beginning over 200 years ago, whereas decreases in the four other countries were inferred to start within the last 200 years. This can be explained by the onset of colonial rule in Africa, when sport hunting became popular in much of sub-Saharan Africa in the latter half of the 19^th^ century (approximately 170 years ago) when European powers were competing for control of the continent’s resources^35^. Similarly, other factors associated with *K*-selected species such as the high mortality of calves and relatively low reproductive rates^36^ have also been responsible for the slow recovery of the species.

By this time, however, South Africa was already a thriving colony for 200 years with much of its wildlife already severely depleted. This is why the historic mtDNA sample from South Africa is old (comprising four of the five oldest samples in our historic data set (see Supplementary Table S1) and small (*n* = 5), because black rhinoceroses were already rare in this country by the mid 1800s when museum collecting became popular. Today, the remnant KwaZulu-Natal population of South Africa comprises over 2,000 individuals, all carrying a single mtDNA haplotype^6^ leading to speculation about whether this extreme loss in genetic diversity indicated a loss of historic diversity or rather reflected historically low levels of diversity in the region^20^. Here, even with a small historic sample, we show that while the historic effective population size in South Africa was among the lowest of the five countries, its pre-bottleneck mtDNA diversity contained six haplotypes (Table 1), which includes a specimen shot in the KwaZulu-Natal province in 1913 whose haplotype is not monophyletic with the present day KwaZulu-Natal haplotype. Namibia also appears to have maintained a lower historic effective population size (Fig. 4c) and genetic diversity, if the genetically indistinguishable historic sample from South-West Angola can be used as a surrogate for Namibia, where historic data are lacking (Tables 1 and 2). This may be related to this population’s comparatively isolated geographic location and/or ecological specialisation which allows South-West African black rhinoceroses to survive in an almost waterless environment.

All five extant populations reached their lowest effective population sizes in the latter part of the 20^th^ century during the upsurge of illegal hunting of rhinoceroses specifically for their horns. The rhinoceros population of Zimbabwe appears to have reached its lowest effective population size in the last 50 years, but has since recovered to a level similar to that of fifty years previously (Fig. 4b). Therefore, although this population has lost genetic diversity, these losses appear only moderate for nuclear and mitochondrial DNA (Tables 1 and 2). This may be credited to the conservation authorities of that country, who recognised the impossibility of protecting the last 300 black rhinoceroses in the Zambezi Valley in the face of unrelenting cross-border poaching from Zambia and Mozambique. These survivors were translocated out of the valley in the 1990s to more defendable areas within Zimbabwe and subsequently underwent high population growth rates^20^, thus moderating the losses of genetic variation due to drift.

We observed the greatest decreases in both effective population size and genetic diversity in East Africa (Tanzania and Kenya). The onset of these losses can be inferred to 100–150 years ago (Fig. 4d and e). Colonial-era British Kenya and German East Africa (Tanzania) represented popular venues for big game hunting, with hunting parties touring the region from the late 19^th^ century^37^,^38^. In addition, much land was cleared of game by authorities seeking to establish agriculture in the newly settled colonies. Yet, large numbers of black rhinoceroses were known in East Africa as recently as the early 1960s^39^. Therefore, the major declines in diversity and effective population size are likely to have resulted mainly from poaching episode that began in Kenya and Tanzania in the early 1980s. Despite this depredation that saw an overall population reduction of 98%^40^, the two East African countries still hold Africa’s most genetically diverse black rhinoceros populations, with the highest inferred mtDNA effective population sizes.

Dividing the sample into respective local populations improved the resolution of geographic patterns of mtDNA loss. Highest haplotype diversity could be localised to the area comprising southern Kenya/northern Tanzania. East African populations also featured the highest levels of haplogroup diversity with three populations harbouring up to three mtDNA haplogroups and one, central Tanzania, up to four. The majority of black rhinoceros fossil data, including the oldest known fossil, also originates in this region^41^, and together with its high genetic diversity suggests East Africa as a putative origin for this species. Other centres of historic haplotype diversity were the Luangwa and Zambezi Valleys in Central southern Africa.

The most severe form of global population extinction in our scheme could be inferred in just under a third of all black rhinoceros populations (11/34 local populations; Fig. 3) and three entire mtDNA haplogroups (WW, NE and RU; Fig. 2a and b), effectively eliminating all genetic variation historically present in West Africa, North-East Africa, parts of Central Africa and the former Cape province (southern part) of South Africa. Genetic extinctions of private haplotypes also occurred in a further five East and South-West African populations, bringing the proportion of mtDNA variation lost solely through population extirpations to 61% (27/44). The remaining 39% of lost historic variation can be attributed to genetic erosion due to genetic drift. Extirpation of seven local populations did not result in the loss of unique mtDNA haplotypes. This is because local extinctions were all within the geographic extent of haplogroups EA and CE north of the Zambezi and SN and SE to the south, and thus shared with populations under more intensive protection in Kenya, Zimbabwe and South Africa. Therefore, the extant populations in these countries may serve as ideal sources for the reintroduction of black rhinoceroses to those places where they have become locally extinct.

We inferred genetic structure from both mtDNA and nuclear microsatellite markers. Differences in evolutionary dynamics and inheritance between these marker types mean that they often do not detect the same patterns of species-wide genetic structure^42^,^43^. Despite this, we were able to infer similar geographic structuring of genetic variation in both marker types. Most conclusively, both markers identified distinct lineages/populations on either side of the Zambezi River, dividing the species similarly into southern African and Central eastern African halves. Furthermore, the geographic ranges of the six genetic populations (SE, SN, SW, EA, CV, CE; Fig. 7) inferred from nDNA overall supported the geographic distribution of similarly named mtDNA haplogroups (Fig. 6). This overlap in the power of marker resolution implies that patterns of gene flow/isolation were similar over extended periods of black rhinoceros evolution. Mitochondrial DNA resolution increased going further back in time, identifying the lineages that diverged early in the species’ evolutionary history. The haplogroups resulting from these early divergences (such as WW and NE) were not distinguishable from microsatellite allele frequencies. This could be because high evolutionary rates and constraints in microsatellite allele size are known to result in high homoplasy^44^,^45^ suggesting that these nuclear markers reached the limit of their evolutionary resolution among the most phylogenetically divergent lineages L1 and L2. On the other hand, microsatellite loci were able to resolve more recently diverged populations (Fig. 5a and b), especially those within southern Africa, where SW and SE mtDNA haplotypes may not have had enough time to assort into distinct population specific lineages (Fig. 2a and b).

Apart from the resolving power of the respective marker types, DNA preservation rates meant that mtDNA could be analysed in a much higher proportion of the historical samples (40%), compared to microsatellites (10%). This is exemplified by the haplogroup RU, where only mtDNA data were available for most of the historic specimens from the region. This may also help explain why the magnitude of erosion of genetic diversity was much greater for mtDNA than for microsatellites. Maternally inherited mtDNA haplotypes are four times more prone to extinction through genetic drift than nuclear alleles. A future examination of our extant and historic genetic data set using the greater number of molecular markers afforded by newer generation sequencing technologies and methods that enable reliable data generation from museum specimens (e.g., enrichment through capture) will undoubtedly shed more light on the structure of genetic variation in this species. This approach will also distinguish between neutral and adaptive genetic variation, aiding in the inference of, and mechanisms underpinning, local adaptaion, thus leading to a more holistic management of this species’ adaptive potential.

Taxonomic delineations based on a limited number of morphological characteristics are often not congruent with molecular genetic structure^46^,^47^,^48^,^49^. In the black rhinoceros, nDNA and mtDNA structure complemented each other, neither mapped clearly onto any of the major competing subspecies definitions based on geographic differences in skull morphology. Zukowsky’s detailed (1964) treatise on morphological affinities across the black rhinoceros distribution remains the most highly resolved taxonomic analysis for this species, and was the only scheme predicting the genetic discontinuity we observed on either side of the Zambezi River. All other major taxonomies invoke a single south-central subspecies (*D. b. minor*), thought to inhabit a massive region including the KwaZulu-Natal province of South Africa in the south and Tanzania in the north, thus spanning the Zambezi. Here we show that this region was historically inhabited by two nDNA populations (SE and SN) to the south and three (CE, EA, CV) to the north of the divide. The geographic isolation of the RU mtDNA haplogroup in the east of this region also suggests additional genetic structuring, however, RU may correspond to *D. b. rovumae* and *D. b. nyasae* from Zukowsky’s (1964)^10^ classification. Similarly, the distinctiveness of DuToit’s^11^ south-western subspecies (*D. b. bicornis*), also known as (*D. b. occidentalis*)^13^ is supported by our SW nDNA population, and the geographic isolation of the SW mtDNA haplogroup to Namibia and the south-western part of Angola. Interestingly, our data set also included a DNA sequence from Sparrman’s Cape rhinoceros Sparrman (1778)^50^, which is an uncontested example of the nominate form *D. b. bicornis* (Rookmaaker and Groves 1978)^14^. This specimen from Kommadagga, and another from what is now the Eastern Cape province of South Africa, both clustered within the spatial distribution of the SE haplogroup, together with historic samples from KwaZulu-Natal, the South African Highveld and the extant KwaZulu-Natal haplotype. Since the interpolated distributions of both the SW nDNA population (Fig. 7) and the SW mtDNA haplogroup (Fig. 6) do not include the type locality for *bicornis* (Cape of Good Hope, south-western part of Western Cape province) the application of the name *bicornis* to the extant Namibian population is erroneous. Several taxonomists recognised the evolutionary distinctiveness of populations in North-eastern and North-western Africa, but only Spinage (1986)^51^ hinted that they may be sister taxa. The now extinct NE haplogroup can be associated with *D. b. brucii*^9^,^13^, but following du Toit (1987)^11^, the AfRSG incorrectly consider the north-eastern black rhinoceros no different to the Kenyan *D. b. michaeli*^40^. Rhinoceroses inhabiting the north-western parts of the species’ range were considered *D. b. longipes*, but here we show strong mtDNA discontinuity across the Shari-Logone River system, with haplogroup WW to the west and haplogroup CV to the east as far as East Africa. If this genetic discontinuity was taken to denote subspecies, then the name *longipes* would correspond to haplogroup CV because it includes a DNA sequence from the type location of this subspecies in Mogrum, which is on the eastern bank of the Shari in Chad.

Limited resources necessitate prioritisation or ranking of management areas for conservation. For the black rhinoceros, even the most comprehensive previous system of priority ranking^52^ could not identify the local populations at risk of undermining the species’ evolutionary potential. This may have been partly because of a focus on populations with larger numbers, but also because the idea of distinctiveness was based entirely on taxonomy.

Here, we applied the genetic ESU/MU concept of Moritz (1994)^29^ and defined the geographic range of these conservation units using spatially informed genetic structuring. Although four of the eight ESUs defined from mtDNA monophyly were geographically distinct, half of them (WW and NE) are already globally extinct and therefore will not be considered further for conservation. This places a high conservation priority to the evolutionarily and spatially distinct populations in Namibia (SW) and Selous Game Reserve in Tanzania (RU). The future of the south-western black rhinoceros looks promising, with high population growth rates it has become even more numerous than the ubiquitous KwaZulu-Natal type^53^. However, this success is partly due to introductions from Namibia to the Northern Cape province of South Africa, where the south-western black rhinoceros has become established extralimitally. In contrast, almost nothing is known about the existence of the small population inhabiting the Selous, but given the recent reports of elephant poaching in Tanzania, it is unlikely that this population still persists. We have therefore listed this population as globally extinct (Fig. 3).

The CV ESU was geographically distinct throughout much of its range, but overlapped in East Africa with CE and EA. It is also in East Africa, specifically in the Masai Mara Game Reserve, where the only extant individual in our data set (MA1516) known to bear the CV haplogroup was observed. The CV nDNA population is also found only in the Masai Mara Game Reserve, but was observed in six individuals (Table S1). It is highly unlikely that more than a handful of this genetic lineage exists, and its success may depend on the management of the small Masai Mara-Serengeti black rhinoceros population.

ESUs EA and CE overlap in Kenya and Tanzania. EA remains in high frequency in both *in situ* and *ex situ* Kenyan populations, so the management of this ESU could be straightforward. In contrast, the geographic distribution of the CE ESU suggests that it would be best managed by conserving what remains of the black rhinoceros population in Tanzania. Although the extant Tanzanian sample was small (*n* = 3), the larger museum sample shows that CE historically occurred at high frequencies in Tanzania and southern Kenya (Fig. 3). However, CE was found in modern day Kenya only in the Masai Mara Game Reserve and Ol Pejeta Conservancy. CE was also observed in two populations descended from individuals captured before Kenya’s 1980s population crash. The first was captured in 1961/1962 from the Makueni district of southern Kenya and moved to Addo Elephant National Park (but subsequently inhabited a private game park in South Africa before being reintroduced into the Mkomazi National Park in Tanzania) and the second in 1967/1978 from Tsavo National Park (now at the Dvůr Králové Zoo, Czech Republic). This suggests that black rhinoceroses across Kenya were not only more diverse, but also more structured historically, and that recent consolidation by the Kenya Wildlife Service from 1963–2008 into several mixed populations, but which excluded the Masai Mara (Muya *et al*.^18^), has resulted in the near loss of the CE haplogroup from the main Kenyan population. Furthermore, the typically KwaZulu-Natal microsatellite profile of one Makueni individual is evidence that this population was not kept as well separated while they were sequestered in South Africa as the public was led to believe. These findings highlight the immense value of *ex situ* captive breeding stocks in Africa, Europe, and potentially in North America, for *in situ* conservation.

Nuclear genetic populations SN and SE were found to be distinct from each other, but mtDNA did not appear to have sorted into monophyletic and geographically distinct ESUs. Kotzé *et al*.^20^ suggested this evidenced a level of historical connectivity between Zimbabwean and South African populations. Therefore, SE could be managed as an MU separately from SN, but new populations geographically intermediate to the current Zambezi and KwaZulu-Natal populations may potentially be seeded with a combined management approach for the SN and SE populations.

In light of the present crisis, conservation priorities should remain the protection and survival of extant populations. It is clear that for the black rhinoceros to have a future in which evolutionary processes can occur, management against the ongoing poaching threat is the top priority. However once the current poaching episode subsides, the genetic management of the remaining, reduced stocks will undoubtedly be a key focus for the long-term survival of the species.

## Methods

### Samples and extractions

Samples were collected from across the species range from a variety of sources (Supplementary Table S1), including universities (mitochondrial DNA (mtDNA) = 80, microsatellite (Msat) genotypes = 37), zoos (mtDNA = 24, Msat = 30), private hunters (mtDNA = 1), museum specimens from collections in Europe (mtDNA = 148, Msat = 42), the USA (mtDNA = 28, Msat = 12) and Africa (mtDNA = 10, Msat = 3) and faecal samples collected in the field by the authors (Msat = 6). We also included previously published data^18^,^20^,^54^,^55^. Specimens in our dataset represented the known species range from Nigeria to Somalia (west – east) and from Eritrea to South Africa (north – south). Chronologically, the samples ranged from one of the oldest specimens collected – Anders Sparrman’s Cape rhinoceros from 1775^50^ to those collected as recently as 2008, (Table S1). Locality information, minimally to country-level, was available or could be deduced from records for all except 41 specimens. Samples from museum collections comprised dried skin, tissue or bone. Samples collected for this study received ethical approval from Cardiff University and were collected in accordance with the protocols/guidelines of the National Zoological Gardens of South Africa (NZG). Where relevant, animals were handled under the guidelines of the American Society of Mammalogists (ASM; Animal Care and Use Committee, 2011)^56^. All permits required at the time of import/export are listed in Supplementary Table S2. All museum samples were collected in accordance with the relevant guidelines and regulations of each museum.

Museum biological samples (250 μg) were rehydrated for 48 hours in distilled water, changing the water once. Bone was ground into a powder. DNA was then extracted according to Rohland *et al*.^57^ in a restricted access ancient DNA laboratory at the Konrad Lorenz Institute in Vienna. All modern samples (blood, skin, tissue and faeces) were extracted using a Qiagen DNAEasy kit with modification for faecal samples.

### Molecular genetics

Mitochondrial DNA was amplified and sequenced for 402 black rhinoceros individuals, including 187 19^th^ and 20^th^ century museum specimens, following Brown and Houlden (2000)^54^ and Anderson-Lederer *et al*.^6^, some with modifications. In addition, nuclear DNA diversity was determined by genotyping 560 individuals (of which 56 were museum specimens) at eight or 11 *D. bicornis*-specific microsatellite loci (see Supplementary Table S3). Full details of the methods are provided in the Supplementary Information.

### Mitochondrial and nuclear genetic diversity

Indices of mtDNA diversity were estimated for three data sets: the total available data, historic (museum) data only, and extant data collected after population declines using Arlequin^58^. Since most historic specimens were collected between 1900 and 1960, haplotypes observed among extant populations must also have been present historically. We calculated levels of diversity for each country and for each of the regional groups recognised by the IUCN’s AfRSG^11^. Multilocus indices of microsatellite genetic diversity were estimated using Arlequin, and allelic richness was corrected for sample size using Adze v. 1.0^59^. Genotyping success rates from museum material were more limited, yielding smaller sample sizes for most countries, and no historic microsatellite data was available from Namibia and South Africa, where museum samples were either very old or absent.

### Genetic erosion

Surviving black rhinoceros populations were hypothesised to have undergone genetic erosion due to genetic drift and/or inbreeding and when local populations have received haplotypes not historically present via translocation. We assessed the level of genetic erosion in surviving stocks by comparing genetic diversity before and after the 20^th^ century population declines. Since all historical material included collection dates, we used coalescent simulation to infer the demographic history of each surviving stock, in order to test whether recorded population reductions translated into a tangible reduction in effective population size over recent time. We ran simulations using a Bayesian extended skyline tree prior^60^ on the entire mtDNA data with tip dates. Simulations were run for 100 million iterations and we scaled the results using a generation time of 24 years, which we calculated as the average of first (5.5 years) and last (42.5) ages of reproduction. We corrected for potential biases due to species-wide population structure^61^ by performing the skyline analysis for each extant stock separately.

### The extinction of local populations

We used genetic information to compile an overview of the change in variation by generating a summary of local populations of black rhinoceroses extant in sub-Saharan Africa based on geographic data from museum specimens and their associated historic information sourced from the invaluable Rhino Resource Centre repository http://www.rhinoresourcecenter.com/62. This assessment allowed the categorisation of the scale of local genetic losses and, because local populations may have inhabited more than one country, enabled us to trace the contribution of each local population to extant country stocks, all of which are consolidations of more than one local population. While some local populations are already well defined, especially in those parts of Africa where conservation authorities promoted or attempted population management (e.g., Zambezi Valley, Sebungwe, KwaZulu-Natal, etc.), the geographic localisation of populations inhabiting other parts of the species’ range were largely unknown. Here we defined a local population by grouping those sampling locations that shared a common geographic descriptor (e.g., southern Tanzania) or inhabited a local area dominated by geological features such as river valleys or basins (e.g., Shari Valley, Victoria Basin). Next, we calculated the historic genetic variation contained within these locations, specifically, the number of mtDNA haplogroups, haplotypes, private haplotypes and the number of haplotypes currently found. This was only possible for the mtDNA data set, as historic nuclear DNA was amplified in fewer samples, resulting in small sample sizes across most of the range. To categorise the genetic effects of 20^th^ century population extinctions, a population was considered locally extinct if all its haplotypes could be detected in other extant populations, whereas it was considered globally extinct if none of its historical haplotypes was sampled among extant populations, that is, extirpation of both the population and all its haplotype variants. Since neither of the above definitions provided a measure of the loss of genetic uniqueness, we further considered an extirpated local population genetically extinct if its private haplotypes did not survive to the present day, even if the shared alleles may still be present in modern day country populations. Lastly, since these surviving stocks were mixed due to population consolidation, we used haplotype sharing data to match genetic persistence of local haplotypes in modern day population stocks.

### Mitochondrial genetic structure

Mitochondrial DNA control region variation was further explored by reconstructing an intraspecific phylogeny from black rhinoceros haplotypes recovered in our data set. Phylogenetic reconstruction was carried out *via* Bayesian coalescent simulation using BEAST^63^ under a Yule model for lineage coalescence and an HKY + G nucleotide substitution model, as determined by model selection in jModelTest v.2^64^. For a full description see the Supplementary Information.

### Nuclear genetic structure

We used Bayesian clustering implemented in STRUCTURE^65^ to examine how microsatellite allele frequencies were structured into discrete populations (*K*) across the species’ range, which in combination with significant allele frequency differences, as measured by population assignment, would in principle confer the inferred populations the status of at least a management unit (MU)^29^. For a full description see the Supplementary Information.

### Spatial analyses and conservation units

Geographic information helps define the spatial scale of genetic units for conservation^25^, and was especially relevant since our range wide analysis brought together genetic data from across most of sub-Saharan Africa. We accounted for this spatial component by incorporating geographic data, where available (mtDNA = 361, nDNA = 560). We conducted spatial analyses separately for both markers using BAPS (Bayesian Analysis of Population Structure) v. 6.0^66^, since this software relaxes the assumption of linkage equilibrium between sites. The spatial model uses molecular data to statistically infer population structure based on a Voronoi neighbourhood system constructed from Dirichlet cells and a hidden Markov random field prior distribution. We explored the patterns of spatial diversity for between one and 20 populations. To visualise the clustering of individuals in a geographic context, we interpolated the posterior probabilities of population membership for both molecular markers across a map of Africa using a restricted maximum likelihood kriging approach (R package, geoR)^67^.

We used the criteria set out by Moritz (1994)^29^ to define ESUs and MUs. The logic behind the scheme is based on levels of inter-population gene flow, as inferred from population structure. Therefore, an ESU is defined as any reciprocally monophyletic mtDNA haplogroup, signifying a lack of long term gene flow. On the other hand, in the absence of mtDNA monophyly, an MU is defined by significant differences in allele frequencies between populations at nDNA (microsatellite) loci. We incorporated the spatial dimension into this scheme by accepting the MU or ESU definition if the geographic distribution of mtDNA haplogroups or nDNA populations were mutually exclusive. In cases of geographical overlap, we collapsed the overlapping haplogroups/populations into higher order ESUs, that is, a monophyletic group containing more than one ESU or MU. We took this approach as some extant populations contain a mixture of members of different mtDNA haplogroups and nDNA populations.

### Data accessibility

Microsatellite data available from the Dryad Digital Repository: http://dx.doi.org/10.5061/dryad.192f1. DNA sequence data available from GenBank (accession numbers KY472315 - KY472605).

## Additional Information

**How to cite this article:** Moodley, Y. *et al*. Extinctions, genetic erosion and conservation options for the black rhinoceros (*Diceros bicornis*). *Sci. Rep.* **7**, 41417; doi: 10.1038/srep41417 (2017).

**Publisher's note:** Springer Nature remains neutral with regard to jurisdictional claims in published maps and institutional affiliations.

## Supplementary Material

Supplementary Information

Supplementary Dataset 1

Supplementary Dataset 2

## Figures and Tables

**Figure 1:**
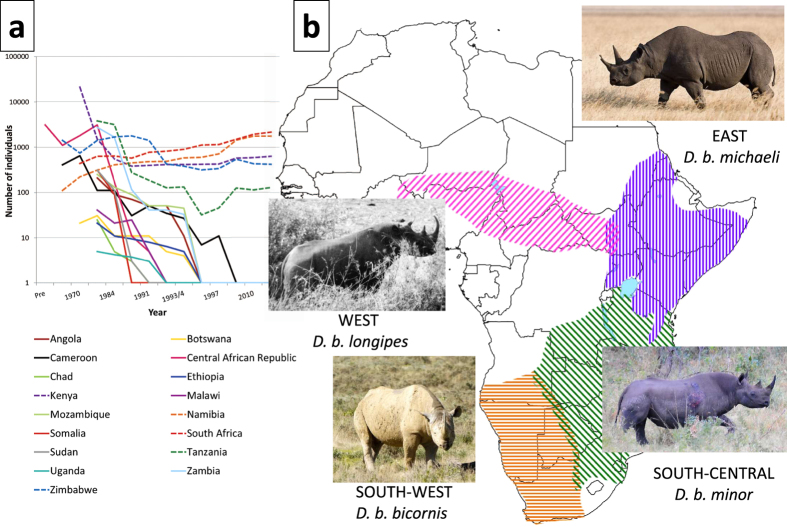
Decrease in black rhinoceros numbers and the distribution of the species across sub-Saharan Africa. (**a**) Decreases in black rhinoceros numbers across Africa in the latter half of the 20^th^ century. Dashed lines indicate extant aboriginal populations. Data from the African Rhino Specialist Group (AfRSG) and (**b**) Range-wide distribution of the black rhinoceros in sub-Saharan Africa. Subspecies mapped are according to du Toit (1987)^11^ and this is the *status quo* for conservation management by the AfRSG. This map was created using the following R packages: geoR^67^, raster^68^, rgdal^69^ and maptools^70^.

**Table 1:** Range-wide mitochondrial control region diversity of black rhinoceroses by country (^#^) and region (^*^).

Species-wide	Total					Historic					Extant				
	*n*	nhaps	P	*h*	pi	*n*	nhaps	P	*h*	pi	*n*	nhaps	P	*h*	pi
	403	64	75	0.96	12.80	146	53	70	0.97	14.64	216	20	41	0.91	8.96
^#^Nigeria	1	1	0	1	0	1	1	0	1	0	Extinct+				
Cameroon	1	1	0	1	0	1	1	0	1	0	Extinct+				
Chad	5	2	4	0.60	2.40	5	2	4	0.60	2.40	Extinct+				
Somalia	6	4	34	0.80	12	6	4	34	0.80	12	Extinct+				
Ethiopia	6	4	19	0.87	7.27	6	4	19	0.87	7.27	Extinct+				
Eritrea	3	1	0	0	0	3	1	0	0	0	Extinct+				
Sudan	3	1	0	0	0	3	1	0	0	0	Extinct+				
Southern Sudan	3	2	3	0.67	2	3	2	3	0.67	2	Extinct+				
Congo (Kinshasa)	2	2	6	1	6	2	2	6	1	6	Extinct+				
Uganda	8	8	23	1	11.96	8	8	23	1	11.96	Extinct+				
Kenya	94	18	31	0.90	6.01	47	16	31	0.90	6.70	47	10	27	0.88	4.99
Tanzania	29	19	36	0.95	8.56	26	19	36	0.96	8.78	3	2	1	0.67	0.67
Mozambique	1	1	0	1	0	1	1	0	1	0	Extinct+				
Malawi	2	2	12	1	12	2	2	12	1	12	Extinct+				
Zambia	8	6	12	0.93	5.25	8	6	12	0.93	5.25	Extinct+				
Zimbabwe	115	8	23	0.76	3.32	6	6	21	0.8	6.98	104	6	8	0.76	2.89
Angola	7	5	23	0.86	10.38	7	5	23	0.86	10.38	Extinct+				
Namibia	31	3	15	0.41	4.37	—					31	3	15	0.41	4.37
Botswana	1	1	0	1	0	1	1	0	1	0	Extinct+				
South Africa	36	6	9	0.31	0.85	5	4	8	0.9	3.8	31	2$	3	0.06	0.19
Unknown Origin	41	23	52	0.94	9.67	41	23	52	0.94	9.67					
^*^West	7	4	27	0.81	12.86	7	4	27	0.81	12.86	Extinct+				
East	146	34	50	0.94	11.56	92	32	49	0.95	13.84	50	11	27	0.88	5.12
South-West	39	7	31	0.53	5.66	8	6	26	0.89	8.86	31	3	15	0.41	4.37
South-central	170	22	42	0.86	5.80	39	22	42	0.96	11.19	135	7	8	0.81	2.96

*n* = number of individuals sampled; nhaps = number of haplotypes; P = number of polymorphic sites; *h* = haplotype diversity; pi = nucleotide diversity.

^$^Additional haplotype introduced from Zimbabwe in the 1970’s.;

^+^population no longer exists.

**Table 2:** Range-wide microsatellite diversity of black rhinoceroses by country (^#^) and region (^*^).

Species-wide	Total					Historic					Extant				
	*n*	loci	*H*_E_	*H*_O_	A	*n*	loci	*H*_E_	*H*_O_	A	*n*	loci	*H*_E_	*H*_O_	A
	560	11	0.62	0.53		56					504	11	0.611	0.53	
^#^Chad	3	8	0.59	0.54	1.59	3	8	0.59	0.54	1.59	Extinct+				
Cameroon	1	11	0.27	0.27	1.33	1	11	0.38	0.38	1.33	Extinct+				
Somalia	3	8	0.60	0.50	1.6	3	8	0.6	0.50	1.6	Extinct+				
Ethiopia	1	8	0.43	0.43	1.33	1	8	0.43	0.43	1.33	Extinct+				
Eritrea	2	7	0.43	0.36	1.39	2	7	0.43	0.36	1.39	Extinct+				
Sudan	1	8	0.75	0.75	1.83	1	8	0.75	0.75	1.83	Extinct+				
Southern Sudan	1	8	0.75	0.75	1.83	1	8	0.75	0.75	1.83	Extinct+				
Uganda	3	8	0.56	0.25	1.56	3	8	0.56	0.25	1.56	Extinct+				
Kenya	180	8	0.69	0.68	1.69	21	8	0.74	0.69	1.78	159	8	0.70	0.70	1.75
Tanzania	11	8	0.75	0.67	1.75	8	8	0.67	0.57	1.75	3	11	0.64	0.70	1.75
Mozambique	1	8	0.75	0.75	1.67	1	8	0.75	0.75	1.67	Extinct+				
Malawi	1	7	0.57	0.57	1.50	1	7	0.57	0.57	1.50	Extinct+				
Zambia	3	8	0.63	0.60	1.63	3	8	0.63	0.60	1.63	Extinct+				
Zimbabwe	242	11	0.52	0.55	1.55	3	8	0.63	0.54	1.73	239	11	0.52	0.55	1.54
Angola	4	8	0.42	0.36	1.42	4	8	0.42	0.36	1.42	Extinct+				
Namibia	31	11	0.49	0.44	1.47	—					31	11	0.49	0.44	1.47
South Africa	72	11	0.45	0.39	1.48	—					72	11	0.45	0.39	1.48
^*^West	4	8	0.71	0.53	1.71	4	8	0.71	0.53	1.71	Extinct+				
East	199	8	0.70	0.66	1.70	37		0.73	0.61	1.73	162	8	0.69	0.70	1.69
South-West	35	11	0.49	0.43	1.47	4		0.42	0.36	1.42	31	11	0.49	0.44	1.47
South-central	322	11	0.54	0.51	1.57	11		0.74	0.58	1.74	311	11	0.53	0.51	1.56

*n* = number of individuals sampled; *H*_E_ = expected heterozygosity; *H*_O_ = observed heterozygosity; A = mean number of alleles per locus (rarefied for sample size).

^+^population no longer exists.

**Table 3:** Defining range-wide conservation units for the black rhinoceros.

mtDNA haplogroups	mtDNA monophyly	Nuclear DNA frequencies different?	Status (Moritz 1994)	Lineage	Spatially distinct?	Higher level ESUs	Extant stock/population
West (WW)	Yes	No	ESU	L1	Yes	ESU	Extinct (no extant populations)
North-East (NE)	Yes	No	ESU	L2a	Yes	ESU	Extinct (no extant populations)
Chari-Victoria (CV)	Yes	Yes	ESU	L2b	Yes	ESU	Kenya (Masai Mara Game Reserve), Tanzania (Serengeti National Park)
Central (CE)	Yes	Yes	ESU	L3a2	No	ESU	Tanzania (all populations), Kenya (Masai Mara Game Reserve)
East (EA)	Yes	Yes	ESU	L3a1	No		Kenya (all populations)
Ruvuma (RU)	Yes	No	ESU	L3b1	Yes	ESU	Likely extinct Tanzania (Selous Game Reserve)
South-western (SW)	Yes (2 lineages)	Yes	ESU	L3b2	Yes	ESU	Namibia; extralimital in South Africa
South-northern (SN)	Yes	Yes	ESU	L3b2	No	ESU	Zimbabwe; extralimital in Kruger National Park
South-eastern (SE)	No	Yes	MU	L3b2	No		South Africa; extralimital in Zimbabwe, Zambia, Botswana, Malawi

Mitochondrial DNA = mtDNA.
