# Supplementary material for: Extinctions, genetic erosion and conservation options for the black rhinoceros (Diceros bicornis)
Source: Sci Rep. 2017 Feb 8;7:41417. doi: 10.1038/srep41417 (PMC5296875; doi:10.1038/srep41417)
Supplement: Supplementary Information [file srep41417-s1.pdf]

SUPPLEMENTARY INFORMATION

Extinctions, genetic erosion and conservation options for the black rhinoceros (*Diceros bicornis*)

Yoshan Moodley, Isa-Rita M. Russo, Desire Lee Dalton, Antoinette Kotzé, Shadrack Muya, Patricia Haubensak, Boglárka Bálint, Gopi K. Munimanda, Caroline Deimel, Andrea Setzer, Kara Dicks, Barbara Herzig, Daniela C. Kalthoff, Hans R. Siegismund, Jan Robovský, Paul O'Donoghue & Michael W. Bruford

Table S2 Permit information for the black rhinoceros samples collected form Namibia and Kenya.

COUNTRY	ORGANISATION	TYPE	NUMBER	HOLDER
United Kingdom	DEFRA	Import	POAO/2010/522	M.W. Bruford
Namibia	Ministry of Environment and Tourism	Collection	1173/2007	P. O'Donoghue
United Kingdom	DEFRA	Import	PATH/22/2007/1	M.W. Bruford
Kenya	CITES Kenya Wildlife Services	Export	004429	M.W. Bruford

Results

Mitochondrial and nuclear genetic structure

Lineages L2 and L3 were subdivided into seven monophyletic haplogroups. Lineage L2 included the haplogroup distributed from the north to the east (NE) of the species' distribution and a haplogroup ranging from east of the Shari-Logone river system to the Victoria Nyanza basin and southern Kenya (CV). The L3 lineage could be divided into sub-lineage L3a (East-central), which contained two haplogroups distributed over a large region from southern Sudan in the north to the Zambezi-Cuando/Chobe Rivers in the south and limited in distribution by the Albertine Rift in the west. Haplogroup EA (East Africa) was represented mostly by Kenyan samples and a Central African haplogroup CE was more commonly found in Tanzania and to the south. Mitochondrial sub-lineage L3b contained all southern African lineages as well as haplogroup RU (Ruvuma), which was limited in distribution to the region between the Shire and Kilombero-Rufiji Rivers. Two haplotypes, although not reciprocally monophyletic, occurred exclusively among specimens from the Damaraland-Koakoland and South-West Angola, west of the Kavango River (coded in orange; Fig. 2A & B). Due to a distinct lack of haplotype sharing between these South-West African black rhinoceroses and those from elsewhere in southern Africa, we considered each South-West African haplotype as a separate monophyletic sub-haplogroup, and refer to both as haplogroup (SW). The only other monophyletic haplogroup in southern Africa was distributed along the lower Kavango/Ngamiland and the region south of the Cuando/Chobe and Zambezi Valleys (SN, South-northern). The remaining southern African haplotypes were polyphyletic and distributed in South-eastern Africa (SE) from the Lower Zambezi River to the southern parts of the former Cape province, and contained both the extant KwaZulu-Natal haplotype and that of Sparrman's Cape rhinoceros (Fig. 2A).

Nuclear DNA variation showed five clusters ($K = 5$). Cluster I was predominantly found at sites within South Africa and showed the least evidence of admixture, as deduced from individual multilocus profiles. Cluster II comprised individuals from Namibia and Cluster III comprised individuals from Zimbabwe. These populations included individuals sampled south of the Zambezi and Cunado/Chobe Rivers, and corresponded roughly to extant black rhinoceros stock populations in northern (Zambezi-Sebungwe), western (Damaraland-Kaokoland) and eastern (KwaZulu-Natal) regions of southern Africa. A fourth, East African group, mainly comprised individuals from Kenya, and a more widespread Central African cluster included samples from countries north of the Zambezi-Cuando/Chobe system (Fig. 4A). Admixture was detected among populations within southern Africa and among populations within Central, North-eastern and North-western and eastern Africa, but not between groups on either side of the Zambezi-Chobe Rivers (Fig. 4B). In general, the nDNA and mtDNA structure were very similar across the species range, with the Zambezi-Chobe Rivers separating three southern African groups (SE, SN and SW) from those in the east (EA) and Central Africa (CE). The only exception being that the CE nDNA population consists of four distinct mtDNA haplogroups (CE, NE, CV and WW). One individual from the small Kenyan population that was relocated to Addo Elephant National Park (Figs. 4A & B) is clearly of South African and not Kenyan origin.

Methods

Molecular genetics

We amplified the 5' end of the control region using primers mt15996L (5'-TCCACCATCAGCACCCAAAGC-3')[S1] and mt16502H (5'-

TTTGATGGCCCTGAAGTAAGAACCA-3')[S2]. These primers were found to align to the *D. bicornis* mtDNA reference genome NC_012682[S3] at positions 15408 and 15939, respectively. This resulted in a 477 bp control region fragment sequenced in 402 individuals, 187 of which were museum specimens and 362 included locality information. Polymerase chain reactions (PCR) was carried out using 50-100ng/µl of DNA in a 25ul reaction containing 1x PCR buffer, 3mM $MgCl_2$, 0.2mM of each DNTP, 0.1 mg/µl purified BSA (New England BioLabs), 0.2µM of each primer and 1.25U Amplitaq Gold DNA polymerase (Promega). Reactions were denatured at 95 ºC for 5 min followed by 45 cycles of 94 ºC for 30 sec, 60 ºC for 1 min, 72 ºC for 1 min, with a final extension of 72 ºC for 10 min. Museum specimens were amplified/sequenced at least twice for consistency. Sequencing reactions were run through an ABI 3130*xl* Genetic Analyser and sequences were assembled, trimmed to 477 bp (removing primer sequences) and aligned in CLC DNA Workbench (CLC Biotech, Qiagen Aarhus). We were able to amplify and sequence the control region fragment consistently in 159 of 217 museum specimens. We also designed internal primers that amplified the region in three smaller overlapping fragments of size 200 bp, using primers mt15996L and Db15608H (5'CTTATATGCATGGGGCAA-3'); 275 bp using Db15564L (5'GGGTATGTATATCGTGCATT-3') and DB15839H (5'AGGATTGATGATTTCCCG-3'); and 154 bp using Db15785L (5'ATCACCACCAATATTCCG-3') and mt16502H. We were thus able to sequence a further 28 samples, bringing our success rate with museum specimens to 86% for mtDNA.

Nuclear DNA diversity was determined by genotyping all available rhinoceros samples. The individuals collected specifically for this study (see Supplementary Table S1 online) were amplified for 11 loci, using 50-100ng/µl of DNA in a 10µl reaction

containing 5µl of QIAGEN Multiplex PCR Master Mix, 0.1µl of 0.001 mg/µl purified BSA (New England BioLabs; 10mg/ml) and 0.2µM of each of the forward and reverse primers. Reactions were cycled at an initial denaturation step at 95 ºC for 15 min, followed by 40 cycles at 94 ºC for 30 sec, a specific annealing temperature (see Supplementary Table S3 online for annealing temperatures) for 1.30 min, 72 ºC for 1 min and a final extension of 72 ºC for 30 min (Simplex 1- DB44; Simplex 2 – DB23; Multiplex 1 – Primers BR17, DB1; Multiplex 2 – Primers BR4, BR6). Primers DB14, B1RH2B, R1RH37D were amplified in a multiplex reaction using a touchdown PCR with an initial denaturation step at 95 ºC for 15 min, followed by 10 cycles of 30 sec at 94 ºC, 30 sec at 48 ºC and 30 sec at 72 ºC; 10 cycles with an annealing temperature at 44 ºC and 20 cycles with an annealing temperature at 40 ºC. The final extension step was done at 72 ºC for 30 min. Primers SW35 and RHI32A were amplified in a second multiplex using the touchdown PCR method described. All alleles were scored by eye in GeneMarker v 1.91 (SoftGenetics LLC) and rechecked by an independent researcher. DNA samples from published specimens were electrophoresed together with samples collected for this study, in order to calibrate electrophoretic differences between compiled data sets. Museum specimens were re-amplified at least three times to confirm allele scores. Loci DB14, B1RH2B and B1RH37D either failed to amplify or amplified inconsistently in museum and faecal samples and were thus omitted for this subset of the data. Our final microsatellite data set comprised 560 individuals, of which 56 were museum samples, genotyped at only eight of the 11 loci. It was known from other studies[S4,S5] that the markers used amplify reliably and without null alleles. Nevertheless, we tested the data generated in this study for scoring errors, allelic dropout and null alleles using MICROCHECKER[S6]. We also tested each pair of loci in our data set for Hardy-Weinberg and genotypic disequilibrium using FSTAT[S7].

Mitochondrial genetic structure

Simulations comprised 500 million MCMC steps, sampling the posterior distribution every 50,000 steps removing the first 20% of the results to ensure we sampled from the stationary part of the distribution. We rooted the phylogeny using homologous sequences of a northern and a southern white rhinoceros (*Ceratotherium simum cottoni* and *C. s. simum* respectively). To further explore our phylogenetic hypothesis, and to ascertain range wide haplotype frequencies, we also constructed an unrooted phylogenetic network, using all 403 control region sequences and invoking the median joining criterion in the software Network v. 4.6.1.2[S8].

Nuclear genetic structure

We carried out five repetitions for each value of *K* between 1 and 10, allowing for individuals with mixed ancestry (admixture model) and with each run comprising two million MCMC (Markov chain Monte Carlo) iterations. We discarded the first 20% of runs as burn-in. We selected the number of populations based on the simulations giving the highest posterior log-likelihood, but accounting for the artefactual introduction of "ghost populations" that are present in the majority of multilocus profiles at low frequency.

References

S1. Campbell, N.J.H., Harriss, F.C., Elphinstone, M.S. & Baverstock, P.R. Outgroup heteroduplex analysis using temperature gradient gel electrophoresis: high resolution, large scale, screening of DNA variation in the mitochondrial control region. *Mol. Ecol.* **4**, 407-418 (1995).

S2. Moro, D., Campbell, N.J.H., Elphinstone, M.S. & Baverstock, P.R. The thevenard island mouse: historic and conservation implications from mitochondrial DNA sequence-variation. *Pac. Conserv. Biol.* **4**, 282-288 (1998).

S3. Willerslev, E. *et al.* Analysis of complete mitochondrial genomes from extinct and extant rhinoceroses reveals lack of phylogenetic resolution. *BMC Evol. Biol.* **9,** 95 (2009).

S4. Muya, S.M. *et al.* Substantial molecular variation and low genetic structure in Kenya's black rhinoceros: implications for conservation. *Conserv. Genet.* **12**, 1575-1588 (2011).

S5. Kotzé, A., Dalton, D., Du Toit, R., Anderson, N. & Moodley, Y. Genetic structure of the black rhinoceros (*Diceros bicornis*) in south-eastern Africa. *Conserv. Genet.* **15**, 1479-1489 (2014).

S6. Van Oosterhout, C., Hutchinson, W.F., Wills, D.P.M. & Shipley, P. MICRO-CHECKER: software for identifying and correcting genotyping errors in microsatellite data. *Mol. Ecol. Notes* **4**, 535-538 (2004).

S7. Goudet. J. FSTAT (Version 1.2): a computer program to calculate F-statistics. *J. Hered*. **86**, 485-486 (1995).

S8. Bandelt, H.J., Forster, P. & Rohl, A. Median-joining networks for inferring intraspecific phylogenies. *Mol. Biol. Evol.* **16**, 37-48 (1999).
